# Continuous Variation Rather than Specialization in the Egg Phenotypes of Cuckoos (*Cuculus canorus*) Parasitizing Two Sympatric Reed Warbler Species

**DOI:** 10.1371/journal.pone.0106650

**Published:** 2014-09-02

**Authors:** Szymon M. Drobniak, Andrzej Dyrcz, Joanna Sudyka, Mariusz Cichoń

**Affiliations:** 1 Institute of Environmental Sciences, Jagiellonian University, Kraków, Poland; 2 Department of Behavioural Ecology, Wroclaw University, Wroclaw, Poland; Hungarian Academy of Sciences, Hungary

## Abstract

The evolution of brood parasitism has long attracted considerable attention among behavioural ecologists, especially in the common cuckoo system. Common cuckoos (*Cuculus canorus*) are obligatory brood parasites, laying eggs in nests of passerines and specializing on specific host species. Specialized races of cuckoos are genetically distinct. Often in a given area, cuckoos encounter multiple hosts showing substantial variation in egg morphology. Exploiting different hosts should lead to egg-phenotype specialization in cuckoos to match egg phenotypes of the hosts. Here we test this assumption using a wild population of two sympatrically occurring host species: the great reed warbler (*Acrocephalus arundinaceus*) and reed warbler (*A. scirpaceus*). Using colour spectrophotometry, egg shell dynamometry and egg size measurements, we studied egg morphologies of cuckoos parasitizing these two hosts. In spite of observing clear differences between host egg phenotypes, we found no clear differences in cuckoo egg morphologies. Interestingly, although chromatically cuckoo eggs were more similar to reed warbler eggs, after taking into account achromatic differences, cuckoo eggs seemed to be equally similar to both host species. We hypothesize that such pattern may represent an initial stage of an averaging strategy of cuckoos, that – instead of specializing for specific hosts or exploiting only one host – adapt to multiple hosts.

## Introduction

The common cuckoo (*Cuculus canorus*) is an obligate brood parasite utilizing a wide range of hosts that differ substantially in body size, egg morphology and reproductive behaviour. Brood parasites usually have strong impacts on host fitness, which induces strong selection pressures on the host to avoid parasites, leading to a co-evolutionary arms race [1]. The common cuckoo has particularly strong effects on its host as the parasite does not allow the host to rear any offspring. Thus, the naive hosts might quickly go extinct if they do not develop antiparasite adaptations. Egg recognition is one of the most prominent antiparasite adaptations. However, the parasite usually develops contradaptation in the form of egg mimicry. Cuckoo's potential hosts, however, have eggs that differ substantially in size, shape and colouration. In response to this diversity, cuckoos have evolved specialization to specific hosts. Within the cuckoo species in Europe, there are about 16 variants (called gentes) of egg types that match the most common host egg phenotypes [2]. Egg-phenotype matching between specific cuckoo gentes and their hosts is remarkable, and in some cases, almost perfect [2]–[5]. Gentes are genetically distinct and are inherited mainly through maternal genetic line [6], [7].

Adaptation to parasitizing specific host species seems unquestionable in cuckoos and is apparent both on genetic and phenotypic levels. However, cuckoo females belonging to one gens may exploit a number of sympatric host species differing substantially in egg phenotypes [8]–[10]. In such a case, cuckoos should either 1) lay eggs that mimic the eggs of only one host species, thus laying dissimilar eggs in nests of other host species – which would be particularly expected in the early stages of a host-parasite arms race [11], or 2) show a generalist strategy by producing eggs dissimilar to eggs of any of the available host species [10]. Either strong specialization to the host species or a generalist strategy of averaging between potential hosts may be favoured by selection in such circumstances. Strong specialization should lead to the evolution of specific phenotypes matching specific host species even within a single gens, maximizing the breeding success of individual cuckoos. The evolution of such discrete egg morphs is supported by field data [12] and numerical simulations, which show that the equilibrium endpoint of an evolving brood parasite – host system is the formation of discrete egg morphs produced by the parasite [11], [13]. Mathematical simulations prevent a continuous distribution of cuckoo egg-morphs from becoming a stable endpoint – discrete egg classes arise even if the initial distribution of egg phenotypes is continuous [11]. However, models allow for a continuous distribution of parasitic egg phenotypes in the initial stages of the evolutionary arms race [11], [13]. Following the introduction of a new host species, producing continuously distributed egg morph, centred around an average phenotype, might be more advantageous in terms of the breeding success than immediate production of specialized eggs – particularly if such specialization requires slow genetic adaptation. Such averaging strategy should result in the disappearance of discrete mimicry to specific hosts, resulting in substantial variation in egg phenotypes observed locally (i.e. regardless of larger-scale patterns resulting from overlapping geographical variation in host egg phenotypes). In both cases, one possible scenario is the evolution of substantial variation in egg phenotype even within a single gens, but specialization should result in discontinuity of the distribution of cuckoo egg phenotypes with regard to the parasitized hosts. One has to also be aware that in the wild population, substantial imbalance in the numbers of available hosts of different species might constraint variation in cuckoo phenotypes: in the case of one prevalent host species and one or more other host species present at substantially lower numbers, cuckoos might evolve egg phenotypes that match the egg phenotype of the most common host [12], [14]. Thus, the nature of observed intra- and inter-gentes variation in egg phenotypes, together with host species frequencies, are important when considering different evolutionary scenarios shaping cuckoo egg mimicry.

Here our goal was to test whether cuckoo eggs found in sympatrically occurring (Eurasian) reed warbler (*Acrocephalus scirpaceus*) and great reed warbler (*A. arundinaceus*) nests differ in size, colouration and the strength of the eggshell. Cuckoos parasitizing these two species are assigned to either the *Sylvia* egg morph or the *Acrocephalus* morph [8], [15], [16]. In the studied population, based on visual identification, both host species are parasitized with the same *Sylvia* morph [17], [18]. Although exploited by one cuckoo egg morph, the eggs of these two host species are clearly distinct in terms of size and coloration. Reed warblers are also less vigilant towards cuckoos than great reed warbles and more readily accept cuckoo eggs [17]. In an egg discrimination experiment carried out in the study population, great reed warblers rejected 13.6% (n = 44 nests) of alien mimetic eggs (an egg of the host species taken from another nest) and reed warbler 3.6% (n = 28) of alien mimetic eggs. Similar statistics calculated for alien non-mimetic eggs (a conspecific egg painted plain brown or blue) showed 92.9% (n = 70) rejections in great reed warbler and 61.8% (n = 204) in reed warbler. Thus, clear differences in egg morphology and anti-parasite behaviour between these two hosts species provide a suitable case to study parasite egg mimicry strategies. In this paper, we try to disentangle whether the cuckoo (i) employs the averaging strategy to match the continuum of egg appearance between these two host species, (ii) shows a better match to one of the species (possibly more frequently utilized) or (iii) exhibits dichotomous specialization by laying eggs matching both host species. In our analysis, we employ morphological measurements, eggshell colour analysis [4], [19], and eggshell strength measurements [20], [21].

## Materials and Methods

### Field methods

The study was conducted from May to August in each of three consecutive years (2011–2013) in the population of two reed warbler species, located at the Stawy Milickie fishponds complex in southern Poland [17], [22]. In this area reed warbler densities reach 57 breeding pairs/ha. We attempted to locate all reed warbler and great reed warbler nests in the area. Their locations were obtained through systematic search in reed-beds. Cuckoo eggs were identified during egg-laying and subsequently collected before hatching and frozen until further analysed. Shells of hatched eggs were also collected if possible. Additionally, in 2012 we collected single eggs from different nests of each of the two host species (15 of great reed warbler and 11 of reed warbler).

### Egg measurements

All collected eggs were thawed and opened after the field season to collect cuckoos' genetic material. Prior to this, we photographed and colour-measured all collected eggs. Identical measurements were also done with respect to host-species eggs. Photographing was done using the Canon EOS 450D camera with a 17–70 mm lens. Photographs were taken at the focal length of approximately 25 mm with automatic shutter and iris settings. Photographs were used solely for measuring physical dimension of eggs. Colour measurements were performed using the Ocean optics JAZ portable spectrophotometer with xenon pulsed-light source (100 pulses per second) and a bifurcating optic fibre. With the probe held perpendicular to the shell surface, we lit the shell surface from a distance of 3 mm (which resulted in a lit area of approx. 3 mm^2^). We measured each egg 12 times at equally-spaced spots on their surfaces. Both photographing and spectrophotometry were performed in different light conditions and at different locations each year, which might contribute to the naturally observed year differences – however all eggs were treated identically within each study year. Since all eggs were measured after the respective field seasons, their colour might change slightly due to chemical degradation of egg-shell compounds [19], [23]. Unfortunately, we cannot assign precise collection rates to the eggs. We assume that the possible effects of prolonged storage (if any) would not be confounded with host-species identities, and thus would not affect subsequent comparisons. Importantly, all eggs were kept frozen in the darkness until assayed, which should further reduce any colour-related deterioration of eggshells.

Reflectance spectra obtained with JAZ were further analysed in the *pavo* package ver. 0.3–1 [24] in R ver. 2.15.0 [25]. After averaging the spectra within each measured egg and smoothing the resulting curves, we analysed resulting spectra using the average bird visual system, employing functions implemented in the *pavo* package. Briefly, individual spectra were transformed into cone receptor quantum catches (*Q_i_*) defined as:
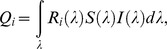
taking into account *i*-th receptor sensitivity, colour reflectance spectrum *S* and illuminant spectrum *I*. Quantum catches of each receptor where then transformed into Cartesian coordinates defining the position of each data point in a tetrahedral colour space spanned on the vertices of a tetrahedron (vertices represent full relative stimulations of red, blue, green and UV receptors). In this representation, the central point of the tetrahedron (equal to [0, 0] of the Cartesian coordinates system) is the achromatic point of black/white colour. Hue in this system is defined using two angles: theta *θ* defined on the RGB plane describes the human-visible part of the hue, whereas phi *φ* represent the deviation in the *z*-axis direction, measuring the UV component [26]. Chroma of the colour can be defined as the Euclidean distance *r* from this achromatic centre. In subsequent analyses, we used both *θ* and *φ* as hue measures, and *r* and achieved *r_a_* (percent of maximum possible chroma for a given hue) as measures of chroma. Additionally, as tetrahedral-space colour model does not provide a measure of brilliance or brightness, we extracted mean brightness for each spectrum, defined as:
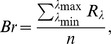
which is the mean reflectance *R* over all *n* recorded wavelengths.

We also used generated quantum catches to analyse differences between eggs according to the receptor noise model, which returns colour distances between measured samples accounting for receptor-related noise in signal perception [27]. Colour contrasts (chromatic and achromatic) were generated for pairs of reed warbler vs. great reed warbler eggs, cuckoo eggs from reed warbler nests vs. cuckoo eggs from great reed warbler nests, and finally for reed warbler/great reed warbler vs. cuckoo eggs. Contrasts are expressed in the units of just noticeable differences (JND, see [24], [27] and were generated assuming four colour receptors (long-, medium-, short- and very-short-wavelengths) with relative receptor densities characteristic for a blue tit retina [5], [24].

Egg photographs were used to obtain their physical measurements (length and width in the widest place). Dimensions were measured to the nearest 0.1 mm in the ImageJ software [28]. These variables were then used in calculations of egg volumes (we used a commonly applied formula: 0.498× length × width^2^ [29]; volume was then expressed in cm^3^).

Additionally, all cuckoo eggs collected in 2011 were subject to shell-strength measurements. Briefly, eggshells were analysed using a pressure-sensitive specimeter that measures force and work required to break the shell. Shell resistance to breaking was analysed at a number of spots (sharp end, blunt end, side of the egg); both inward and outward breaking force and work were considered. Respective parameters were measured to the nearest 0.001 N (force) and 0.00001 J (work).

### Statistical analyses

All measured variables for cuckoo eggs were analysed using a simple linear model with year and host species as fixed effects (*lm* procedure in R). Egg-thickness measures (available only for one year) were analysed using a two-tailed Student *t* test (*t.test* procedure in R). In all tests *α* = 0.05 was used as the acceptable threshold type-I error. Prior to analyses, all variables were centered and standardized to have zero mean and unity variance. We ensured that all fitted models returned homoscedastic and approximately normal residuals.

All measured colour variables together with egg width, length, volume and shape, transformed for normality if necessary (see above) and standardized, were aggregated into principal components using the *prcomp* procedure in R. The first two components were used to represent each egg in a multivariate colour space to depict the presence or absence of clustering of eggs within the groups of two host species. This procedure was applied both to the cuckoo eggs and host species eggs for comparison. The resulting graph depicts the joint variation of reed warbler and cuckoo eggs in all measured variables. Additionally we provide a projection plot depicting positions of all eggs in the tetrahedral colour space projected on the circumscribed sphere of the tetrahedron. To visualize variation in hues/chromas and areas of overlapping colours, we generated in *pavo* convex hulls encapsulating respective groups of points in the tetrahedral colour space. We provide both numerical outputs from these analyses (proportions of overlapping hulls) and graphical depictions.

In total we collected and measured 15 cuckoo eggs from great reed warbler nests and 47 cuckoo eggs from reed warbler nests. Twelve eggs (great read warbler – 4, reed warbler – 8) were measured for shell-thickness.

### Ethics statement

This study was carried out under the license granted by the division of the Polish Bioethical Committee at Wroclaw University (to AD). The license covered collection of the eggs, egg measurements and subsequent procedures. All procedures were performed so that they minimized the stress exhibited by birds during egg sampling and monitoring.

## Results

**Figure 1:**
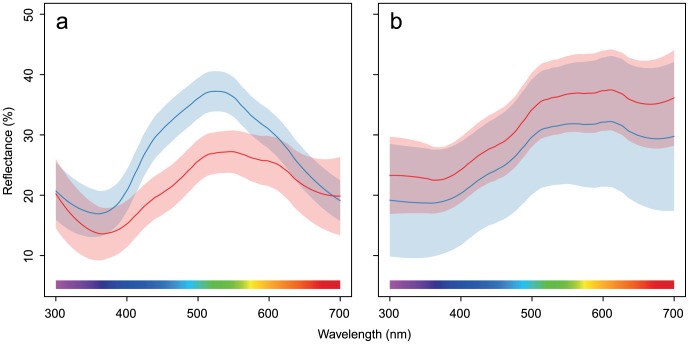
Average smoothed reflectance spectra of warbler eggs (a) and cuckoo eggs (b). Blue lines: eggs from great reed warbler nests; red lines: eggs from reed warbler nests. Bands represent 95% confidence regions.

We found no differences in shell strength in cuckoo eggs collected in nests of the two host species (Table S1). In particular, we found no significant differences in either the mean inward or outward breaking force (inward: *t_df = 6.81_* = 0.13, *P* = 0.9; outward: *t_df = 5.01_* = 0.48, *P* = 0.65). Eggs laid by cuckoos into nests of the two host species did not differ in their shape index (one means perfect sphere): *t_df = 59_* = 0.01, *P* = 0.98. Overall, size variables and colour measures did not indicate any differentiation (Table 1, Figure 1a).

**Table 1:** Comparison of differences in colour-related and size-related variables for cuckoo eggs laid in nests of reed warblers (rw) and great reed warblers (grw).

Variable name	Trait value ± SE (rw)	Trait value ± SE (grw)	t	P
*Br*	31.00±2.54	26.31±3.91	0.99	0.32
*θ*	2.80±0.04	2.81±0.06	0.20	0.84
*φ*	−0.48±0.06	−0.51±0.09	0.25	0.80
*r*	0.12±0.01	0.11±0.01	0.55	0.59
*r_a_*	0.35±0.02	0.35±0.04	0.07	0.94
Length	22.10±0.16	22.20±0.16	0.28	0.78
Width	16.54±0.12	16.66±0.12	0.49	0.62
Volume	3.02±0.05	3.08±0.05	0.48	0.63

Estimates were obtained from linear models and are corrected for the effect of study years. All tests are performed with *df* = 59.

**Figure 2:**
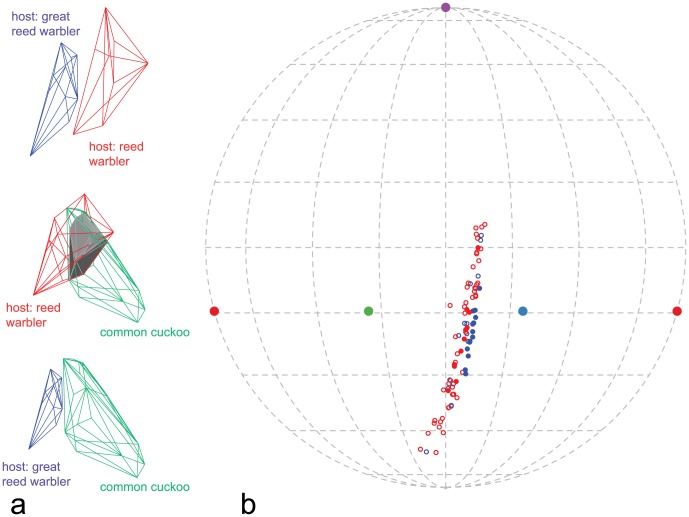
Cuckoo and host eggs in tetrahedral bird average visual colour space. (a) Overlap of convex hulls (grey polyhedron) encapsulating point measurements of host (red and blue) and cuckoo (green) eggs. Second and third plot are presented in identical orientations for ease of comparison and rotated to maximize the visibility of differences on the third plot (i.e. where there is no overlap). (b) Projection of colour measurement on a circumscribed sphere of the colour space tetrahedron; open symbols – cuckoos, filled symbols – hosts, red – reed warbler nests, blue – great reed warbler nests.

Host species significantly differed with respect to several egg-morphology variables. We found significant differences in shell brightness (*t_df = 20.76_* = 2.67, *P* = 0.01), RGB hue *θ* (*t_df = 12.48_* = 3.77, *P* = 0.002), realized chroma *r* (*t_df = 17.68_* = 5.65, *P* = 0.005), and achieved chroma *r_a_* (*t_df = 15.57_* = 2.47, *P* = 0.03); no significant differences in UV hue *φ* were found (*t_df = 15.44_* = 0.34, *P* = 0.73). Great reed warbler eggs are, on average, brighter, more saturated in the blue range, and generally lack the red hue (Figure 1b). Moreover, compared to reed warbler eggs, they are longer (*t* = 9.97, *df* = 19.60, *P*<0.0001) and wider (*t* = 11.38, *df* = 21.49, *P*<0.0001), which also translates into larger volume (*t* = 14.29, *df* = 21.99, *P*<0.0001). Colour differences are also apparent in the tetrahedral colour space. The two host species clearly occupy separate clusters (Figure 2, Table 2), and the convex hulls surrounding colour measurements do not overlap.

**Table 2:** Summary of colour variables measured in the tetrahedral colour space for three analysed species.

Species	*h_m_*	mean *r_a_*	Convex hull overlap [%] with		
			*A.arundinaceus*	*A.scirpaceus*	*C.canorus*
*A.arundinaceus*	0.033	0.390	–	–	–
*A.scirpaceus*	0.047	0.325	0	–	–
Cuckoo	0.071	0.351	0	47	–

For each species we present: mean hue span (*h_m_*), average saturation (mean *r_a_*), and overlap of convex hulls calculated as: *volume_of_overlap*/*volume_of_smaller_hull* (thus the value of 1 would indicate perfect inclusion of the smaller hull in the bigger one; see [24] for more details).

Cuckoos' eggs occupy the space between the two host species, although they seem to group closer to the reed warbler eggs. This indicates that cuckoo eggs are closer to reed warbler eggs in terms of hue and chroma. This is further confirmed by a substantial overlap of convex hulls of cuckoo and reed warbler eggs (Table 2, Figure 2). In contrast, regions occupied by cuckoos' and great reed warblers' eggs do not overlap (Figure 2), however they are very close to each other. For clarity we did not present separate analogous analyses of overlap for cuckoos split by the host species – they occupy virtually the same region in the colour space and overlap extensively (over 90%).

**Figure 3:**
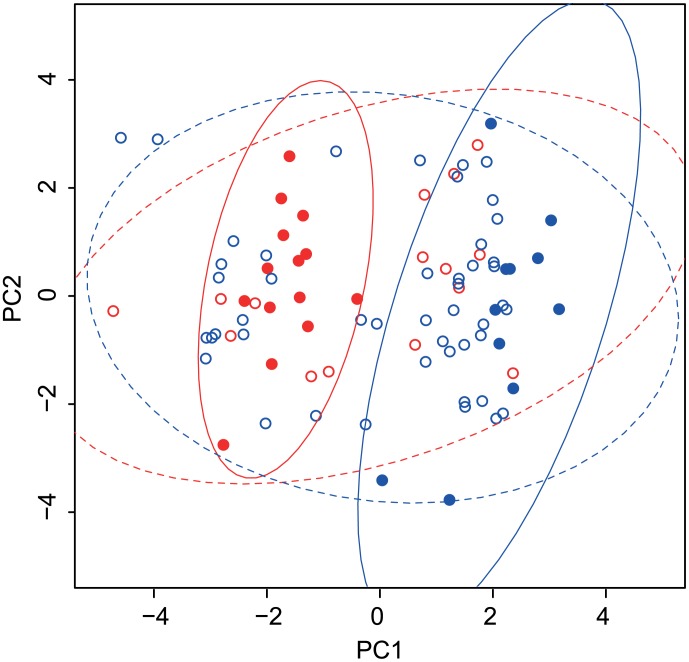
The first two principal components describing colour and size of cuckoo and warbler eggs. Blue symbols - great reed warbler eggs (filled) or cuckoo eggs found in great warbler nests (open); red symbols - reed warbler eggs (filled) or cuckoo eggs from reed warbler nests (open). Lines represent bivariate 95% confidence ellipses. Line colours correspond to circle colours. Solid lines - host species; dashed lines - cuckoos.

Tetrahedral colour space representation does not account for differences in brightness. We have thus represented all measured eggs using principal components derived using all colour (i.e. hue, chroma and brightness) and size variables. Differences between host species are apparent after representing all measured host species eggs on the plane defined by the first two principal components (Figure 3). The two host species form two clearly separated clusters of points indicating they are clearly differentiated. An analogous plot representing all measured cuckoo eggs (colour and size variables) indicates that they form a uniform, non-differentiated population of points, with the variation among cuckoo eggs being substantially larger than within host species and clearly overlapping with variation in both host species (Figure 3). The first principal components represent mainly physical dimensions, chroma and RGB hue, whereas second components represent mainly brightness, RGB and UV hues (see Table S2 for loadings of all variables on the first two components). The first principal component explained 51% of total variance in eggs' morphology and the second component explained 34% of variance. Correlations between original variables included in the PCA are provided in Table S3.

A receptor-noise model (Vorobyev & Osorio) further confirmed the above observations (Table 3). The average chromatic contrast between cuckoo eggs and great reed warbler was similar to the chromatic contrast between great reed warbler and reed warbler. On the other hand, the achromatic contrast between cuckoo eggs and either of the two host species appeared to be similar in value and slightly higher than the contrast between the host species. Both chromatic and achromatic contrasts between cuckoo eggs found in nests of reed warbler and great reed warbler were not significantly higher than contrasts calculated within the host-species groups (all P>0.05), supporting a lack of any differentiation among cuckoo eggs.

**Table 3:** Receptor-noise model output for chromatic and achromatic contrasts between cuckoos and host species.

Comparison	Chromatic contrast	Achromatic contrast
Host eggs		
GRW – RW	1.23±0.05	6.05±0.29
GRW – GRW	0.58±0.06	3.43±0.40
RW – RW	0.90±0.08	4.95±0.49
Cuckoo eggs		
CC_RW – CC_GRW	1.67±0.05	2.61±0.07
CC_RW – CC_RW	1.27±0.02	2.09±0.04
CC_GRW – CC_GRW	1.27±0.21	2.07±0.31
Host eggs vs. cuckoo eggs^1^		
CC – GRW	1.28±0.01	6.26±0.09
CC – RW	0.97±0.01	6.32±0.06

All values are in the units of just-noticeable differences (JND) and are presented with respective standard errors. Shortcuts: RW – reed warbler, GRW – great reed warbler, CC – common cuckoo (all eggs), CC_GRW – cuckoo eggs from great reed warbler nests, CC_RW – cuckoos eggs from reed warbler nests.

1 Cuckoo eggs exhibited no significant differences in terms of respective chromatic and achromatic contrasts and thus all cuckoo eggs were compared with respective host species eggs.

## Discussion

Our study confirms clear differentiation in egg appearance between reed warbler and great reed warbler. The eggs differ in size and colour spectra forming two distinct clusters in the principal-components analysis. Despite these clear differences between hosts, we failed to show clear clustering among cuckoos parasitizing these two species with respect to their egg morphologies. A similar conclusion comes from a study performed on four sympatrically occurring warbler species in which no differences in egg colouration were found between cuckoo eggs from nests of those host species [8]. More importantly, our data show that cuckoo eggs exhibit substantial variability that – in specific morphology components – largely overlaps the variance observed in both species. Interestingly, this overlap is less apparent when analysing only hue and chroma. In terms of chromatic components of egg colouration, all sampled cuckoo eggs were more similar to reed warbler host eggs than to great reed warbler eggs. However, achromatic colour components of cuckoo eggs not only exhibited larger variation, but they also overlapped both host species egg phenotypes. To our knowledge this is the first observation of such variation overlapping multiple hosts in the cuckoo brood parasitic system.

These results clearly indicate that sampled cuckoo eggs comprised phenotypes closely matching both host species as well as a range of intermediate phenotypes. This is very interesting since the proportion of parasitized nests clearly differs between these two host species, so one may expect cuckoo eggs to show a better match to the more frequently utilized host species. During the study, only 5.8% of nests of great reed warbler were parasitized while 11.7% of reed warbler nests were parasitized. Moreover, our long-term observations suggest that great reed warbler has started to be utilized as a potential host only recently. Cuckoo eggs were found in only 2 out of over 700 nests of great reed warbler recorded in the years 1970–2006 [18]. In 2007 – out of 154 nests, 4 contained cuckoo eggs (2.6%); in 2009 – out of 136 nests, 6 cuckoo eggs were found (4.4%). In 2012–2013, 20 cuckoo eggs were found in 356 nests of the great reed warbler (5.6%). Although these proportions are similar to some other European populations (e.g. 5.3% in Moravia (Czech Republic); [30]), they are at the same time in contrast to figures from a number of other locations (Hungary and Bulgaria: 64% and 40% nests parasitized, respectively [7], [31]). These figures indicate that host switching may be an underestimated but important phenomenon in brood-parasites occurring in areas where several suitable hosts are present. Cases of host-switching have rarely been reported (but see Jelinek et al. 2014) – clearly, more research is required in this matter as host switching may represent an important component of continuously an evolving host-parasite system of brood-parasitic species. Our colour measurements are consistent with the process of cuckoos slowly adapting to the newly acquired host species – interestingly, this adaptation seems to occur firstly in achromatic components of colouration. The picture of some components of egg morphology specializing and acquiring mimicry faster than the others is an important indication that egg phenotypes are multivariate entities, with their individual components undergoing partly independent evolutionary changes. Obviously our data lack the dynamic aspect as the egg morphologies have not been tracked in the past. However, the existence of partial specialization in some morphology components and its complete absence in the others suggests that our system may represent and early stage of acquiring a new host, which would be consistent with patterns of egg-morph variability predicted by numerical models [11], [13]. Whether the observed patterns represent a genuine snapshot from a dynamic trajectory of egg-colouration traits evolving in response to host-switching remains to be confirmed. It is possible, that the observed variation is a part of a larger pattern of egg morphological variation. Numerical simulations indicate that host egg morphologies can fluctuate and follow a cyclic patterns changing relatively rapidly [32]. It is possible that similar dynamics occur in co-evolving brood parasites; long term tracking of egg morphologies in brood parasitic systems should allow these exciting questions to be answered. Importantly such tracking would not only put evolving parasitic eggs in the appropriate temporal scale – it would also reveal how parasites shape the evolution of host eggs. It has been recently shown, that egg mimicry can have profound effects on the evolution of host egg phenotype [33]–[35]. In our system a substantial difference exists between the two close cuckoo hosts – further, long-term studies are needed to see how these differences are maintained and evolve in the absence of cuckoo specialization.

The sole presence of substantial variance in cuckoo egg phenotypes suggests that this trait has large evolutionary potential. Rapidly evolving egg mimicry has been recently shown among cuckoos parasitizing reed warblers in Zealand (Denmark) [36]. The authors observed a significant increase in similarity of egg coloration of the cuckoo and their reed warbler host over a 24-year study period. However, the substantial variance in cuckoo egg phenotypes may constitute an adaptation on its own. It is possible that benefits related to the ability to exploit other, less typical host species may overcome any potential costs of failure due to imperfect egg mimicry.

Our analyses focus on size and background colouration of cuckoo eggs, ignoring possible differences in the spotting pattern of cuckoo eggs. However, studies published to date have usually demonstrated background colouration to be the most important trait differentiating gentes [4], [37]. In contrast, studies considering spottiness of eggs failed to show any differences between gentes [36], [38]. Similarly, Antonov et al. [39] found no significant differences in egg spotting between cuckoos parasitizing different hosts, in spite of clear differences in the analysed host species (great reed warbler, marsh warbler and corn bunting). We believe that ignoring egg spottiness in our study does not significantly bias our inference.

Lack of any differentiation in cuckoo egg phenotypes with respect to two parasitized host species was also apparent in the case of shell strength. It is well known that cuckoo eggs are equipped with strong egg shells; a number of hypotheses have been suggested to explain this observation [20], [21], [40]. One hypothesis is that stronger shells protect cuckoo eggs from being punctured and ejected from host nests. In our study, we predicted that cuckoo eggs laid in nests of great reed warblers would have stronger shells, which would be an adaptation to parasitizing hosts with larger and stronger beaks. We found no evidence of such differentiation. The sample size used for shell thickness measurements was relatively small, however given observed effect sizes, it is unlikely that lack of observed differentiation is a matter of power. Also, in this study we used dynamometry, whereas in a number of studies egg shell thickness was measured as a reliable proxy of egg shell strength [41], [42]. In our opinion, the dynamometric approach is more appropriate as it more directly relates measured characteristics to the way the egg shell affects host anti-parasite responses and cuckoo hatching processes [20], [21]. Lack of observed differences between cuckoos parasitizing hosts that differ greatly in their egg puncturing potential may on one hand suggest that the puncture-resistance hypothesis is not adequate in this system. On the other hand, it is possible that eggshell thickness is less evolutionarily labile than egg colouration and does not differentiate so easily with respect to parasitized hosts.

Another potentially confounding aspect of our study which cannot be properly accounted for is the identity of cuckoo females. Obviously, eggs laid by the same females should be expected to be more similar to each other than to eggs laid by other unrelated females. This issue would be particularly important in our study if specific females laid eggs in nests of different host species. Cuckoo females are believed to be specific with respect to chosen host-species although there are rare cases of females laying eggs in nests of multiple host species [1]. Unfortunately, genetic material gathered in this study does not allow for robust and confident identification of cuckoo eggs coming from specific females: the uncertainty of assigning parents deteriorates rapidly if neither of the parental genotypes is known. However, GPS coordinates collected for all sampled nests indicate that the average distance between sampled host nests in this study was 9 km. According to Davies (2011) [1] (see also [43]), the (mostly non-overlapping) breeding areas of female cuckoos range from 0.21 to 1.68 km^2^, resulting in area radii ranging from 250 to 730 m. Even after assuming 730 m as a conservatively large territory size and excluding from the analysis all nests involved in pairs with nest-to-nest distances less than 2·730 = 1460 m (9 nests), we did not find evidence for the presence of significant differentiation in egg phenotypes of cuckoos laying eggs in nests of different hosts. Thus, we assume that our analyses are robust despite the lack of female identities.

Finally, potential bias in our analyses may also be related to egg collection procedure. It is well recognized that hosts tend to reject more dissimilar eggs at higher rates [44], [45], and thus dissimilar eggs might be underrepresented in our data set if such cuckoo eggs disappeared prior to egg collection. This problem should be accounted for while interpreting the data, but it is extremely difficult to validate this source of bias. This would be particularly important if we find that the host species showing higher rejection rates matches cuckoo eggs better that the host species showing lower rejection rates. In fact, our data show a rather opposite pattern, so this potential bias is unlikely to alter our interpretation.

In conclusion, our data do not support the hypothesis of specialization of the common cuckoo in parasitizing two species of reed warblers. In the studied population, cuckoos parasitizing two studied hosts seem to lay phenotypically indistinguishable eggs. Our results directly contrasts with other published studies looking at cuckoos exploiting sets of sympatric hosts [5], [36]. We failed to find significant differences in egg morphology between cuckoos parasitizing these two species despite clear differentiation between host species. Instead, observed colouration patterns strongly depend on the colour components studied. Chromatic components clearly cluster cuckoo eggs close to the reed warbler eggs, supporting the expectation that cuckoos should match most efficiently the most prevalent host species in a given area. However, achromatically, cuckoo eggs exhibit much more variation that spreads continuously between achromatic components of egg morphology of both host species. Finally, as indicated by the subspaces occupied by cuckoo egg phenotypes in the tetrachromatic colour space, ample amount of chromatic variation in cuckoo eggs spreads in a direction opposite to either of the two available host species. The cuckoo from this gens may exploit other host species present in the study area, and this may explain this additional variation in cuckoo egg morphology. Further studies should focus on analysing cuckoo egg phenotypes found in other sympatrically occurring host species. It may be particularly interesting to explore selection regimes that may lead to homogenising of cuckoo egg phenotypes even in the presence of clearly distinct host species egg phenotypes. The studied population is a dynamic system, where host species frequencies undergo continuous changes. Our study demonstrates that in colouration studies, a multivariate approach is the one that yields more realistic results. Ignoring size and brightness in our study would clearly favour greater similarity of cuckoo eggs to those of the reed warbler. However, after including brightness and physical dimensions, the variation in egg phenotypes spreads much wider and may overlap phenotypic values of the other host species. Future studies should look more closely at such patterns. In particular, it seems important to establish the relative importance of different aspects of egg morphology (size, brightness, saturation, hue) in the evolution of host egg discrimination and parasite egg mimicry. The amount of observed differentiation may depend on the choice and assembly of characteristics used in such studies. Different aspects of egg morphology may evolve at different rates in brood-parasitic species, substantially biasing simple univariate comparisons of specific traits.

## Supporting Information

Table S1 **Detailed results of shell-thickness measurement showing differences between host-species tested with a Student t test.**
(DOCX)

Table S2 **Loadings on the first two principal components from all colour and size variables.**
(DOCX)

Table S3 **Correlations of raw variables used in the PCA analysis for hosts (a) and cuckoos (b).**
(DOCX)
