# Supplementary material for: Continuous Variation Rather than Specialization in the Egg Phenotypes of Cuckoos (Cuculus canorus) Parasitizing Two Sympatric Reed Warbler Species
Source: PLoS One. 2014 Sep 2;9(9):e106650. doi: 10.1371/journal.pone.0106650 (PMC4152305; doi:10.1371/journal.pone.0106650)
Supplement: Table S1 — Detailed results of shell-thickness measurement showing differences between host-species tested with a Student t test. (DOCX) [file pone.0106650.s001.docx]

Table S1. Detailed results of shell-thickness measurement showing differences between host-species tested with a Student t test. F – force measures; W – work measures; S, BP, SP – side of the egg, blunt end, sharp end, respectively; i – inward breaking force/work (i.e. from the outside of the egg); o – outward breaking force/work. Forces measured in Newtons [N]; works measured in Joules [J].

| Parameter | *t* | *df* | *P* |
| --- | --- | --- | --- |
| FBPi | 0.51 | 9.9 | 0.62 |
| WBPi | 0.53 | 5.6 | 0.61 |
| FSPi | 1.10 | 9.3 | 0.31 |
| WSPi | 1.80 | 7.6 | 0.10 |
| FSi | 1.96 | 6.0 | 0.09 |
| Wsi | 1.04 | 4.8 | 0.35 |
| FBPo | 0.34 | 8.9 | 0.73 |
| WBPo | 0.37 | 5.6 | 0.72 |
| FSPo | 0.13 | 9.0 | 0.90 |
| WSPo | 0.37 | 5.61 | 0.72 |
| FSo | 0.53 | 4.8 | 0.61 |
| WSo | 0.32 | 7.2 | 0.76 |
