# Supplementary material for: Continuous Variation Rather than Specialization in the Egg Phenotypes of Cuckoos (Cuculus canorus) Parasitizing Two Sympatric Reed Warbler Species
Source: PLoS One. 2014 Sep 2;9(9):e106650. doi: 10.1371/journal.pone.0106650 (PMC4152305; doi:10.1371/journal.pone.0106650)
Supplement: Table S2 — Loadings on the first two principal components from all colour and size variables. (DOCX) [file pone.0106650.s002.docx]

Table S2. Loadings on the first two principal components from all colour and size variables. Variable names are explained in the “Materials and methods” section.

|  | Host species eggs | |
| --- | --- | --- |
|  | PC1 | PC2 |
| Br | -0.16 | 0.53 |
| θ | -0.27 | 0.46 |
| Φ | -0.02 | 0.56 |
| r | -0.37 | -0.35 |
| r_a_ | -0.34 | -0.23 |
| Length | -0.46 | 0.01 |
| Width | -0.45 | 0.002 |
| Volume | -0.47 | 0.02 |
