# Supplementary material for: Continuous Variation Rather than Specialization in the Egg Phenotypes of Cuckoos (Cuculus canorus) Parasitizing Two Sympatric Reed Warbler Species
Source: PLoS One. 2014 Sep 2;9(9):e106650. doi: 10.1371/journal.pone.0106650 (PMC4152305; doi:10.1371/journal.pone.0106650)
Supplement: Table S3 — Correlations of raw variables used in the PCA analysis for hosts (a) and cuckoos (b). (DOCX) [file pone.0106650.s003.docx]

Table S3. Correlations of raw variables used in the PCA analysis for hosts (a) and cuckoos (b).

a)

|  | Br | Theta | Phi | r | r(achieved) | Length | Width | Volume |
| --- | --- | --- | --- | --- | --- | --- | --- | --- |
| Br | 1.00 | 0.81 | 0.80 | -0.31 | -0.12 | 0.32 | 0.28 | 0.32 |
| Theta | 0.81 | 1.00 | 0.75 | 0.04 | 0.15 | 0.50 | 0.47 | 0.51 |
| Phi | 0.80 | 0.75 | 1.00 | -0.52 | -0.23 | -0.08 | -0.10 | -0.07 |
| r | -0.31 | 0.04 | -0.52 | 1.00 | 0.89 | 0.65 | 0.63 | 0.64 |
| r(achieved) | -0.12 | 0.15 | -0.23 | 0.89 | 1.00 | 0.55 | 0.50 | 0.53 |
| Length | 0.32 | 0.50 | -0.08 | 0.65 | 0.55 | 1.00 | 0.87 | 0.94 |
| Width | 0.28 | 0.47 | -0.10 | 0.63 | 0.50 | 0.87 | 1.00 | 0.98 |
| Volume | 0.32 | 0.51 | -0.07 | 0.64 | 0.53 | 0.94 | 0.98 | 1.00 |

b)

|  | Br | Theta | Phi | r | r(achieved) | Length | Width | Volume |
| --- | --- | --- | --- | --- | --- | --- | --- | --- |
| Br | 1.00 | 0.81 | 0.86 | -0.82 | -0.74 | -0.01 | -0.02 | -0.02 |
| Theta | 0.81 | 1.00 | 0.90 | -0.74 | -0.75 | -0.13 | 0.05 | -0.02 |
| Phi | 0.86 | 0.90 | 1.00 | -0.81 | -0.73 | -0.06 | 0.09 | 0.04 |
| r | -0.82 | -0.74 | -0.81 | 1.00 | 0.95 | 0.11 | -0.08 | -0.03 |
| r(achieved) | -0.74 | -0.75 | -0.73 | 0.95 | 1.00 | 0.09 | -0.03 | 0.01 |
| Length | -0.01 | -0.13 | -0.06 | 0.11 | 0.09 | 1.00 | 0.14 | 0.54 |
| Width | -0.02 | 0.05 | 0.09 | -0.08 | -0.03 | 0.14 | 1.00 | 0.91 |
| Volume | -0.02 | -0.02 | 0.04 | -0.03 | 0.01 | 0.54 | 0.91 | 1.00 |
